# Effect of dipeptidyl-peptidase-4 inhibitors on C-reactive protein in patients with type 2 diabetes: a systematic review and meta-analysis

**DOI:** 10.1186/s12944-019-1086-4

**Published:** 2019-06-18

**Authors:** Xin Liu, Peng Men, Bo Wang, Gaojun Cai, Zhigang Zhao

**Affiliations:** 1 Department of Pharmacy, Beijing Tiantan Hospital, Capital Medical University, No 119 South 4th Ring West Road, Fengtai District, Beijing, 100070 China; 2 Department of Pharmacy, Peking University Third Hospital, Beijing, 100191 China; 3 Department of Traditional Chinese Medicine, Liaoning University of Traditional Chinese Medicine, Shenyang, 110032 Liaoning Province China; 4 Department of Cardiology, Wujin hospital affiliated with Jiangsu University, Changzhou, 213017 Jiangsu Province China

**Keywords:** Dipeptidyl peptidase-4 inhibitors, C-reactive protein, Type 2 diabetes mellitus, Randomized controlled trials

## Abstract

**Background:**

Dipeptidyl peptidase-4 inhibitors (DPP-4i) are emerging glucose-lowering agents through interacting with DPP-4 substrate, impact of which on systemic inflammation in type 2 diabetes mellitus (T2DM) remains unknown. This study aimed to evaluate the effect of DPP-4i on modulating serum levels of C-reactive protein (CRP) in T2DM.

**Methods:**

PubMed, Cochrane library and Embase databases were searched. Randomized controlled trials (RCTs) with comparators were selected. A random-effects model was used for quantitative data analysis. Heterogeneity was evaluated with *I*^*2*^ index. Sensitivity analysis was performed using the one-study remove approach.

**Results:**

Sixteen trials with 1607 patients with T2DM were included. Pooled analysis of DPP-4i demonstrated a significant decrease in serum CRP concentrations (− 0.86 mg/L, 95% CI, − 1.36 to − 0.36). No significant difference was found between DPP-4i and active comparators on serum CRP concentrations (0.64 mg/L, 95% CI, − 0.10 to 1.37). Pooled analysis proved to be stable and credible by sensitivity analysis. In subgroup analysis, changes in serum concentrations of CRP were significantly associated with short diabetes duration (− 0.23 mg/L, 95% CI, − 0.41 to − 0.05).

**Conclusions:**

DDP-4i effectively reduced serum CRP levels and showed no stronger effect than traditional oral antidiabetic agents.

International Prospective Register for Systematic Review (PROSPERO) number: CRD42017076838.

**Electronic supplementary material:**

The online version of this article (10.1186/s12944-019-1086-4) contains supplementary material, which is available to authorized users.

## Background

Type 2 diabetes mellitus (T2DM) is a chronic metabolic disorder with decreased insulin action and hyperglycemia [1, 2]. T2DM and insulin resistance (IR) are also increasingly recognized as a chronic inflammatory state like atherosclerosis [3]. Meanwhile, persistent low-grade inflammation leads to peripheral IR and alleviating of inflammatory process improves IR and glucose handling [4].

As inflammation plays a key role in IR, T2DM and cardiovascular disease (CVD), a rational search for inflammatory markers was performed for a better prediction for CVD risks [5]. Among current downstream markers of inflammation, C-reactive protein (CRP) is a sensitive and dynamic protein for predicting systemic inflammation. It is an acute-phase protein synthesized by the liver, concentrations of which increase by up to 10,000-fold during acute responses to serious infection or major tissue damage. CRP was initially found to be negatively correlated with circulating insulin [6]. It was determined by high sensitivity ELISA kits and utilized to reflect acute inflammatory state in clinical practice. Whether or not CRP can be altered by dipeptidyl peptidase 4 inhibitors (DPP-4i) in T2DM remains uncertain.

Inflammation has been recognized as a major risk factor for T2DM. Systemic inflammation is often observed in overweight and obese subjects and elevated CRP concentrations are present in certain 27.6% among this group. Obese subjects are more likely faced with higher levels of CRP compared to normal-weight controls [7]. Serum baseline levels of CRP are markedly higher in patients with diabetes or glucose intolerance [8, 9]. Meta-analysis also shows that higher levels of CRP are significantly positively correlated with increased risk for T2DM [10].

In fact, CRP also plays a significant role in coronary heart disease. CRP binds to low-density lipoprotein cholesterol (LDL-C) and is present in atherosclerotic plaques. This acute-phase protein has been proved to serve as a better predictor for cardiovascular risk than LDL-C [11]. Drugs that reduce CRP concentrations effectively inhibit atherosclerosis progress, especially in patients with diabetes or IR. Thiazolidinediones (TZDs) have been recognized to reduce CRP levels at the molecular and serum levels as useful glucose-lowering agents with off-target or unwanted side effects [12, 13].

DPP-4i improve glycemic control via preventing the inactivation of incretins, such as glucagon-like peptide-1 and glucose dependent insulinotropic peptide [14, 15]. DPP-4i improve pancreatic β-cell function in both fasting and postprandial states in T2DM [16, 17]. In spite of the known efficacy on glucose metabolism, the shape of association between DPP-4i and CRP concentration in T2DM has not been well characterized. Based on the related risk of diabetes and coronary heart disease, CRP might be potentially recognized as a novel surrogate cardiovascular indicator and biomarker for antidiabetic agents. Therefore, the present meta-analysis aimed to help judge the extent to which DPP-4i modulated CRP concentrations in patients with T2DM.

## Methods

### Search strategy

A systematic review was performed according to PRISMA guidelines from Cochrane handbook. Multiple databases including PubMed, Cochrane Library and Embase databases were comprehensively searched. Medical subject heading terms and keywords used to identify studies included: (“C-reactive protein” OR CRP OR “high sensitivity C-reactive protein” OR hsCRP OR hs-CRP) AND (sitagliptin OR vildagliptin OR teneligliptin OR saxagliptin OR linagliptin OR anagliptin OR alogliptin). Randomized controlled trials published in English were identified up to December 31, 2017.

### Study selection

Trials were combined and duplicates were discarded. Studies were first screened on the basis of title and abstract, after which total article was reviewed. Data from the published English languages were extracted. Trials must meet these inclusion criteria: (1) randomized controlled studies compared DPP-4i with current treatment; (2) results reporting CRP levels with DPP-4i treatment; (3) studies conducted in T2DM patients and not in healthy volunteers; and (4) studies were published as full-text articles. Studies were excluded if they were animal studies, narrative reviews, poorly described and only abstract papers. Studies were also excluded if they did not meet criteria listed before. The reference list of eligible articles was hand-searched and corresponding authors were contacted if missing information or clarification was relevant. For duplicate publications from the same study, only the most complete reports were identified. Inclusion and exclusion criteria were evaluated objectively by two reviewers.

### Data extraction

Two reviewers entered the extracted data onto a standardized designed form and summarized the important information. Detailed data of first author, publication year, country origin, sample size, ratio of men and women, body mass index, mean age, diabetes duration, medication intervention, therapy duration and serum CRP concentrations at baseline in each included study were recorded. Studies with different treatment duration were extracted as the longest therapy duration. Related adverse events were summarized according to the information from the identified studies. Main investigators were contacted for missing data. Alterations of CRP concentrations before and after treatment were recorded for analysis. Standard errors and confidence intervals (CIs) were converted into standard deviations (SD) [18]. Otherwise, values would be imputed by assuming SDs of the missing outcome to be the mean of the SDs from the trials that reported relevant information.

### Quality evaluation

The quality of studies was evaluated according to the Cochrane Reviewers’ Handbook [19].The parameters applied for the evaluation of each trial were as follows: random sequence generation, allocation concealment, blinding of participants and personnel, blinding of outcome assessment, incomplete outcome data, selective outcome reporting, and other potential sources of bias. According to the Cochrane criteria, a judgment of ‘yes’ indicated low risk of bias, while ‘no’ indicated high risk of bias. An item of ‘unclear’ indicated an unknown or unclear risk of bias.

### Statistical analysis

Statistical analysis was conducted using STATA 12.0 software. Change scores for serum concentrations of CRP were calculated as follows: value at the end of treatment period – value at baseline. According to whether or not significant heterogeneity of outcomes was present, the continuous variable was pooled as weighted mean difference (WMD) and 95% confidence interval (CI) with a fixed-effects or random-effects model. Heterogeneity among the studies was assessed using a Chi-squared test and quantified with *I*^*2*^ index. Sensitivity analysis was conducted with the leave-one-out method to assess the influence of each study on the overall effect size. Publication bias was examined by Begg’s test and Egger’s test if there were at least five studies for each outcome in the meta-analysis. Additionally, subgroup analysis was performed according to diabetes duration, race, dose, age, CRP and HbA1c at the baseline.

## Results

### Flow of included studies

**Fig. 1:**
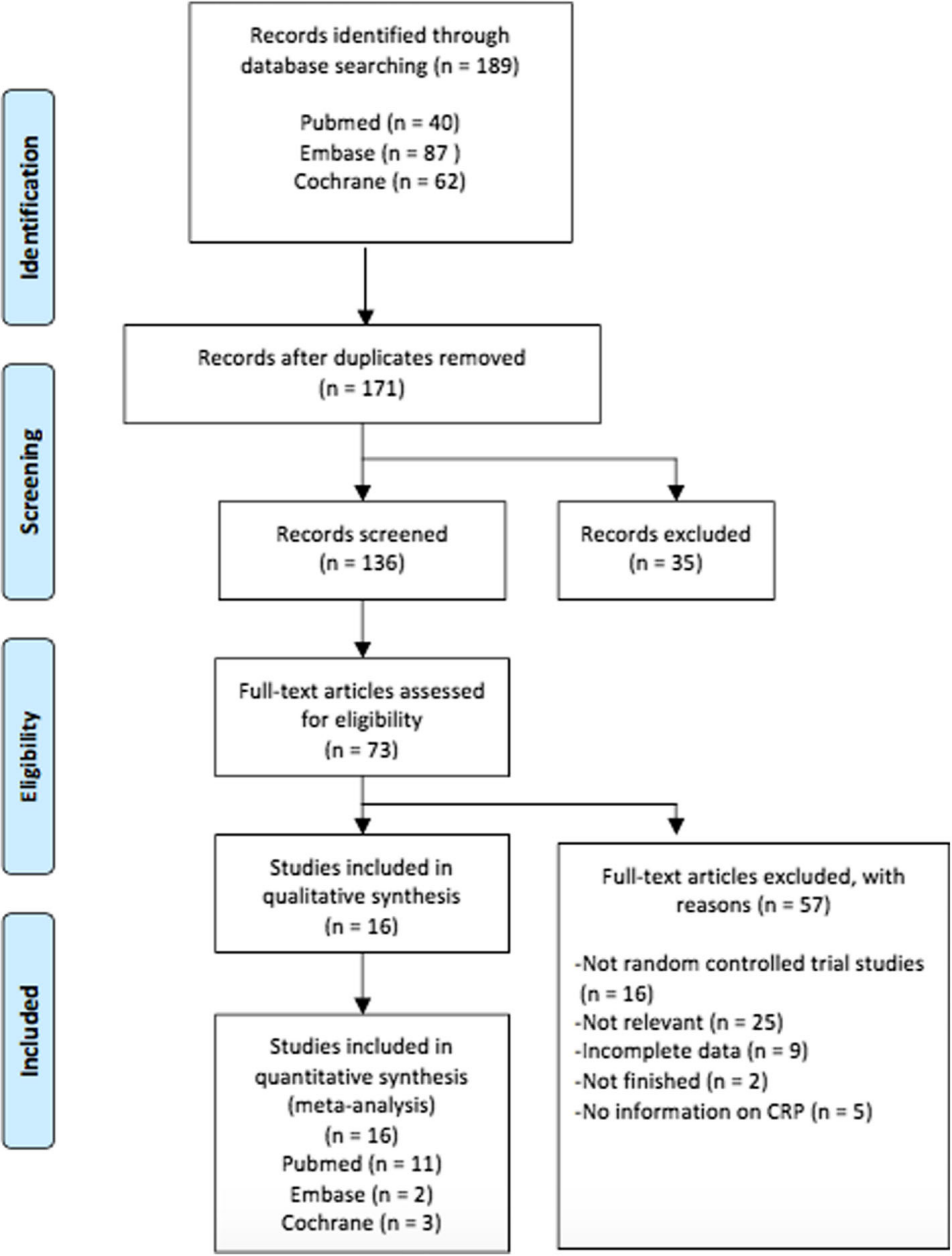
Flow chart of the number of studies identified and included into the meta-analysis

The initial literature search identified 189 records. After removal of inadequate studies, 16 randomized controlled trials with 1607 subjects were eligible for quantitative meta-analysis. Patients with CVD were not included for analysis on the basis of CRP fluctuation in pathological state. Flowchart of inclusion and exclusion was shown (Fig. 1).

### Characteristics of included studies

Baseline characteristics of participants in identified studies were fully presented in Table 1. The largest study had a size of 341 subjects, while the smallest one recruited 25 subjects. Most patients among identified studies received DPP-4i treatment of sitagliptin and vildagliptin. Only two studies compared linagliptin and alogliptin with placebo and traditional antidiabetic agents, respectively. Therapy duration ranged from 3 to 26 months. Different trials conducted by the same researcher were analyzed respectively.

**Table 1:** Demographic characteristics of the studies included

Study, year	Location	Group: dose(n)	HbA1c (%)	Sex (M/F)	Age (years)	BMI (kg/m^2^)	Diabetes duration (months)	Treatment duration (months)	CRP^a^ (mg/L)
Derosa,2012a [20]	Italy	sita:100 mg + met:2500 ± 500 mg(91)	sita+met:8.1 ± 0.8	sita+met:42/49	sita+met:55.9 ± 8.8	sita+met:28.1 ± 1.2	sita+met:5.8 ± 2.6	12	sita+met:-0.5 ± 0.54
		met:2500 ± 500 mg(87)	met:8.0 ± 0.7	met:44/43	met:54.8 ± 7.9	met:28.9 ± 2.0	met:5.4 ± 2.3		pla:-0.4 ± 0.73
Asahara,2013 [21]	Japan	sita:50 mg(24)	sita:8.3 ± 0.2	sita:11/13	sita:62.0 ± 2.3	sita:25.5 ± 0.9	NS	3	sita:-0.3 ± 0.1
		pla:50 mg(24)	pla:8.2 ± 0.2	pla:14/10	pla:58.0 ± 2.4	pla:26.5 ± 0.7			pla:-0.1 ± 0.1
Suzuki,2014 [22]	Japan	sita:50 mg(16)	IG:9.1 ± 1.6	sita:9/7	sita:56.1 ± 15.3	sita:26.3 ± 7.2	sita:22.8 ± 27.6	6	sita:-0.1 ± 0.6
		lira:0.9 mg(24)	lira:9.8 ± 2.2	lira:15/9	lira:58.6 ± 15.9	lira:28.2 ± 7.2	lira:28.8 ± 33.6		lira:-1.9 ± 0.6
Liu,2013 [23]	Taiwan	sita:100 mg(60)	sita:8.27 ± 0.86	sita:22/38	sita:60.1 ± 8.9	sita:26.6 ± 4.6	sita:93.6 ± 51.6	6	sita:-0.07 ± 0.04
		piog:30 mg(60)	piog:8.54 ± 0.97	piog:23/37	piog:58.1 ± 8.3	piog:25.7 ± 3.7	piog:93.6 ± 46.8		piog:-0.19 ± 0.04
Derosa,2010 [24]	Italy	sita:100 mg + piog:30 mg(75)	sita+piog:8.5 ± 0.9	sita+piog:37/38	sita+piog:57.0 ± 5.0	sita+piog:27.9 ± 1.5	sita+piog:5.0 ± 2.0	12	sita+piog:-0.7 ± 0.8
		met:850 mg + piog:30 mg(76)	met+piog:8.4 ± 0.8	met+piog:39/37	met+piog:58.0 ± 6.0	met+piog:27.7 ± 1.3	met+piog:6.0 ± 3.0		met+piog:-0.7 ± 0.71
Nakamura,2014 [25]	Japan	sita:50 mg(24)	sita:7.04 ± 0.56	sita:10/14	sita:66.6 ± 11.9	sita:27.8 ± 3.5	sita:57.6 ± 41.2	3	sita:-1.0 ± 3.6
		vog:0.6 mg(31)	vog:6.94 ± 0.45	vog:18/13	vog:68.4 ± 9.2	vog:25.7 ± 4.3	vog:41.9 ± 44.1		vog:1.2 ± 5.6
Derosas,2012b [26]	Italy	vild:100 mg + met:2500 ± 500 mg(84)	vild+met:8.1 ± 0.6	vild+met:42/42	vild+met:54.2 ± 8.3	vild+met:27.9 ± 1.5	vild+met:6.1 ± 3.7	12	vild+met:-0.8 ± 1.4
		Pla + met:2500 ± 500 mg(83)	pla + met:8.2 ± 0.7	pla + met:43/40	pla + met:52.4 ± 7.1	pla + met:27.8 ± 1.4	pla + met:6.3 ± 3.9		pla + met:-0.4 ± 0.7
Strozik,2014 [27]	Poland	vild:100 mg + met:1500 mg(15)	vild+met:8.2 ± 0.2	vild+met:10/5	vild+met:45.9 ± 4.6	vild+met:28.2 ± 1.8	vild+met:24~60	3	vild+met:-0.49 ± 0.2
		met:1500 mg(13)	met:8.0 ± 0.6	met:9/4	met:51.4 ± 7.2	met:29.0 ± 3.5	met:24~60		met:-0.06 ± 0.3
Zografou,2015 [28]	Greece	vild:50 mg + met:850 mg(32)	vild+met:8.1 ± 0.8	vild+met:18/14	vild+met:52.0 ± 11.2	vild+met:31.6 ± 4.6	NS	6	vild+met:-0.4 ± 2.8
		met:850 mg(32)	met:8.0 ± 0.8	met:20/12	met:56.0 ± 10.5	met:32.2 ± 5.9			met:1.1 ± 1.6
Derosa,2014 [29]	Italy	vild:100 mg(86)	vild:7.9 ± 0.9	vild:42/44	vild:59.8 ± 9.9	vild:27.9 ± 1.6	vild:6.9 ± 4.7	6	vild:-0.4 ± 0.6
		glim:6 mg(70)	glim:7.8 ± 0.8	glim:36/34	glim:56.8 ± 8.9	glim:27.7 ± 1.3	glim:6.7 ± 3.5		glim:-0.3 ± 0.6
Kim,2017 [30]	Korea	vild:50 mg(14)	vild:7.2 ± 0.2	vild:9/5	vild:59.9 ± 10.2	vild:25.8 ± 2.7	NS	4	vild:-0.18 ± 1.1
		piog:15 mg(11)	piog:7.4 ± 0.4	piog:4/7	piog:52.1 ± 11.1	piog:27.4 ± 4.3			piog:-0.55 ± 0.9
Mita,2015 [31]	Japan	alo:25 mg(172)	alo:7.3 ± 0.8	alo:101/71	alo:64.4 ± 9.8	alo:24.6 ± 4.3	alo:9 ± 3.3	26	alo:0.06 ± 0.1
		con:25 mg(169)	con:7.2 ± 0.8	con:98/71	con:64.8 ± 9.1	con:24.9 ± 3.7	con:8.2 ± 3.7		con:0 ± 0
Boer,2017 [32]	Netherland	lin:5 mg(22)	lin:6.3 ± 0.4	lin:13/9	lin:63 ± 4.7	lin:32.3 ± 3.5	lin:18 ± 20	6.5	lin:-0.2 ± 0.7
		pla:5 mg(22)	pla:6.2 ± 0.5	pla:14/8	pla:62 ± 4.3	pla:29 ± 2.3	pla:12 ± 13		pla:0.5 ± 1.0
Yamada,2017 [33]	Japan	sita:50 mg(55)	sit:7.0 ± 0.6	sit:38/17	sit:69 ± 8	sit:25.9 ± 3.3	NS	24	sit:-0.06 ± 0.28
		con:50 mg(60)	con:6.9 ± 0.5	con:39/21	con:69 ± 9	con:24.8 ± 3.9			con:-0.09 ± 0.46
Koren,2012 [34]	Israel	sita:100 mg(20)	NS	NS	NS	NS	NS	3	sita:0.9 ± 1.9
		glib:5 mg(20)							glib:0.29 ± 1.7
Nogueira,2014 [35]	Brazil	sita:100 mg(18)	sit:8.0 ± 0.6	sit:9/9	sit:55.1 ± 6.7	sit:26.5 ± 2.7	sit:130.8 ± 69.6	6	sit:-0.7 ± 2.8
		insu:11 ± 6.7(17)	insu:8.1 ± 0.7	insu:6/11	insu:58.4 ± 6.9	insu:27.5 ± 2.5	insu:130.8 ± 90		insu:-0.1 ± 2.2

Values are expressed as mean ± SD

*Abbreviations*: *n* Number of participants per group, *HbA1c* Glycated haemoglobin, *CRP* C-reactive protein (high sensitivity assay), *sita* Sitagliptin, *vild* Vildagliptin, *alo* Alogliptin, *metf* Metformin, *pla* Placebo, *con* Conventional treatment, *lira* Liraglutide, *piog* Pioglitazone, *vog* Voglibose, *glim* Glimepiride, *lin* Linagliptin, *glib* Glibenclamide, *insu* Insulin, *cos* Chitosan oligosaccharide, *NS* Not stated

^a^CRP parameter presented as the mean change from baseline

### Quality evaluation

Study quality was critically evaluated based on the scheme suggested by the Cochrane criteria. All the studies were randomly designed and details on the items of bias criteria among included trials were summarized in Table 2. Four studies might have detection bias on the basis of blinding of outcome assessment. Additionally, eight trials had performance bias due to absence of implementation of blind methods.

**Table 2:** Risk of bias assessment in the studies identified for meta-analysis

Study, year	Random sequence generation	Allocation concealment	Blinding of participants and personnel	Blinding of outcome assessment	Incomplete outcome data	Selective reporting	Other bias
Derosa, 2012a [20]	L	U	L	L	L	L	L
Asahara, 2013 [21]	L	U	H	L	L	L	L
Suzuki, 2014 [22]	L	U	H	H	L	L	L
Liu, 2013 [23]	L	U	H	H	L	L	L
Derosa, 2010 [24]	L	U	L	L	L	L	L
Nakamura, 2014 [25]	L	U	U	U	L	L	L
Derosas, 2012b [26]	L	U	L	L	L	L	L
Strozik, 2014 [27]	L	L	U	U	L	L	L
Zografou, 2015 [28]	L	U	U	U	L	L	L
Derosa, 2014 [29]	L	U	L	L	L	L	L
Kim, 2017 [30]	L	U	H	H	L	L	L
Mita, 2015 [31]	L	U	H	L	L	L	L
Boer, 2017 [32]	L	U	L	L	L	L	L
Yamada, 2017 [33]	L	U	H	L	L	L	L
Koren, 2012 [34]	L	U	H	H	L	L	L
Nogueira, 2014 [35]	L	U	H	L	L	L	L

Criteria defined for quality assessment are based on the Cochrane guidelines

*Abbreviations*: *H* High risk of bias, *L* Low risk of bias, *U* Unclear or unrevealed risk of bias

### Meta-analysis of the impact of DPP-4i treatment

**Fig. 2:**
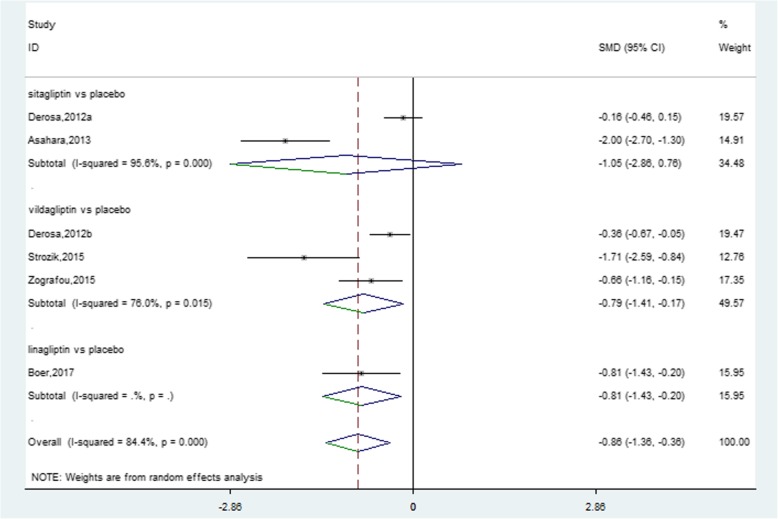
Forest plot for the impact of DDP-4i treatment versus placebo on CRP concentrations

**Fig. 3:**
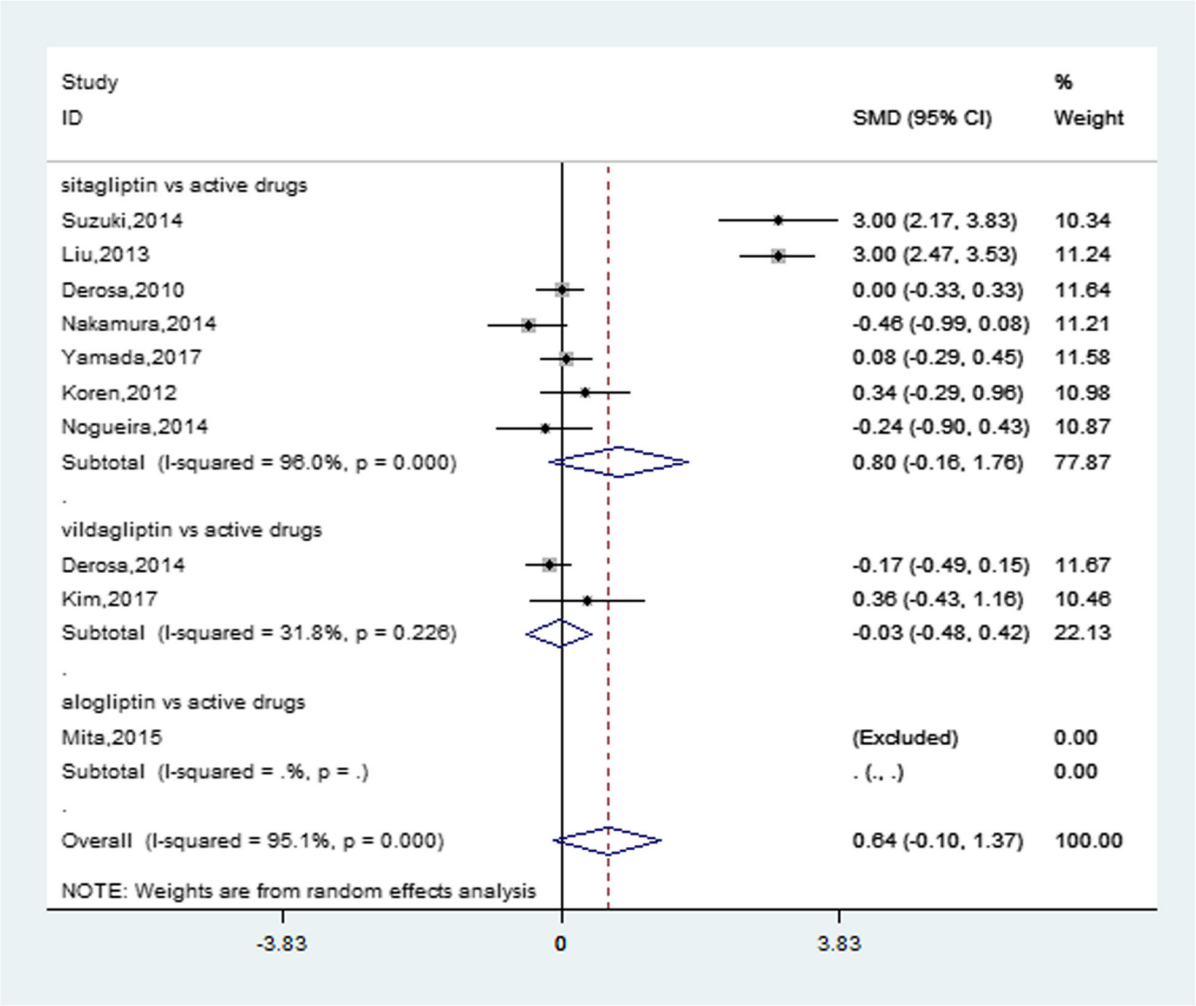
Forest plot for the impact of DDP-4i treatment versus active comparator on CRP concentrations

Meta-analysis demonstrated that DPP-4i lowered CRP concentrations compared to placebo by − 0.86 mg/L (95% CI, − 1.36 to − 0.36, *P* = 0.001) (Fig. 2) and a significant heterogeneity was observed between these studies (*I*^*2*^ = 84.4%). Due to different chemical structure, sitagliptin and vildagliptin might have different efficacy on reducing serum CRP levels. Vildagliptin further reduced CRP concentrations by − 0.79 mg/L (95% CI, − 1.41 to − 0.17, *I*^*2*^ = 76%, *P* = 0.013) compared to sitagliptin (− 1.05 mg/L, 95% CI, − 2.86 to 0.76, *I*^*2*^ = 95.6%, *P* = 0.254). However, DPP-4i showed no stronger impact on reducing serum CRP levels than traditional antidiabetic agents and significant heterogeneity occurred between studies (0.64 mg/L, 95% CI, − 0.10 to 1.37, *I*^*2*^ = 95.1%, *P* = 0.090) (Fig. 3).

**Fig. 4:**
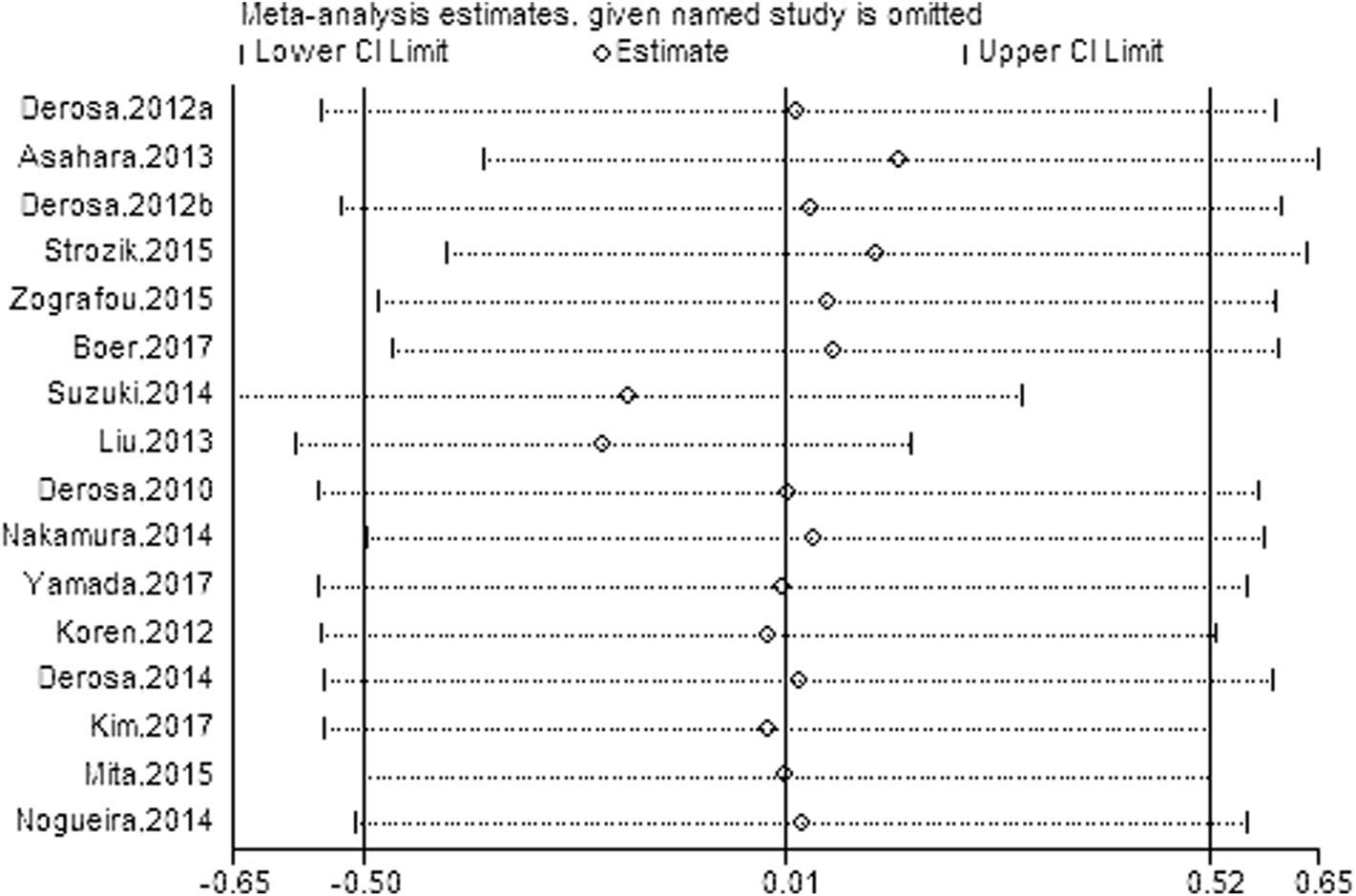
Leave-one-out sensitivity analysis for the impact of DPP-4i on serum concentrations of CRP

**Fig. 5:**
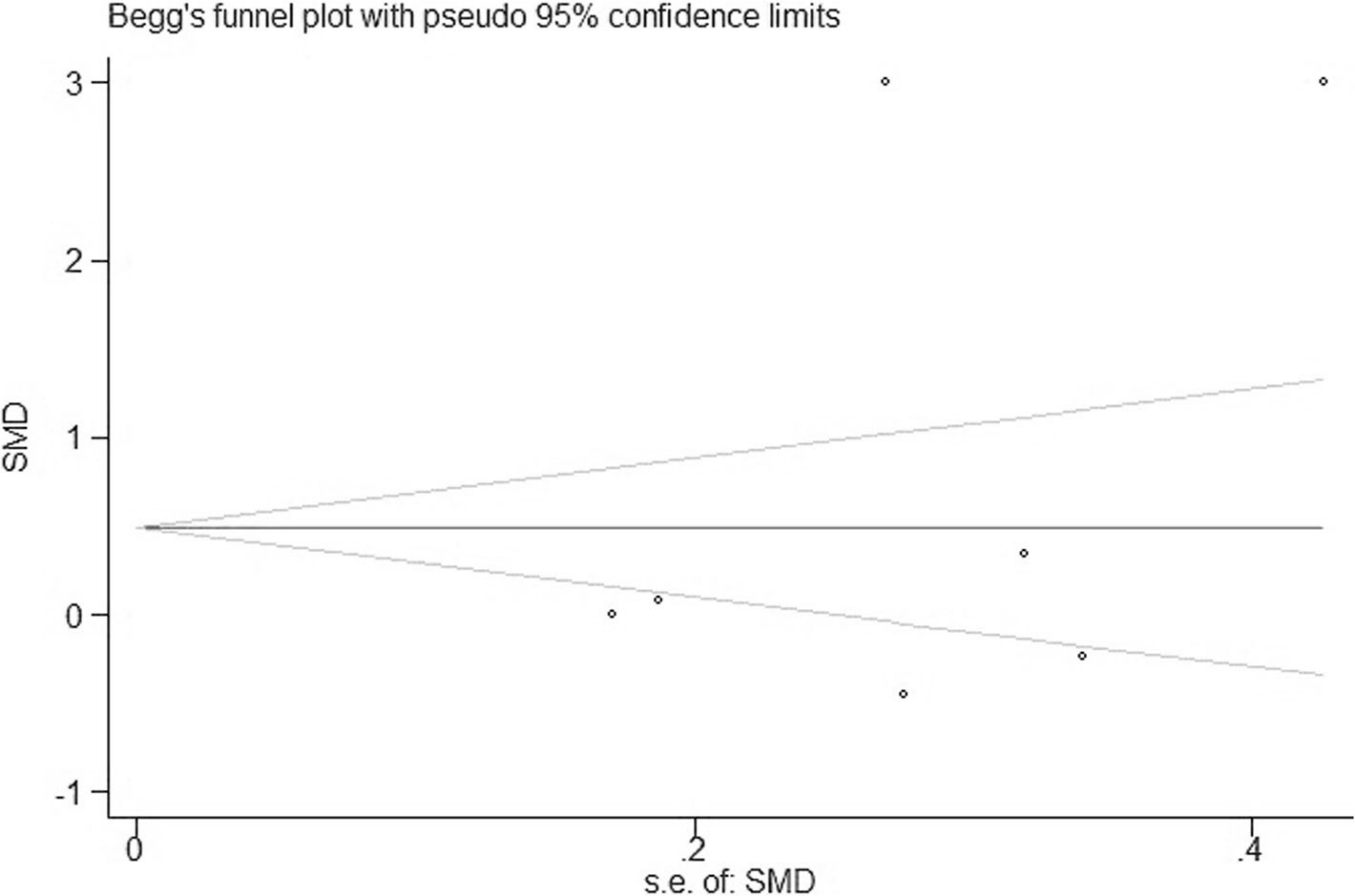
Assessment of publication bias in the meta-analysis of studies reporting the impact of DPP-4i on serum concentrations of CRP

Sensitivity analysis was performed to validate these results, illustrating that the pooled results were stable and credible (Fig. 4). In the sensitivity analysis, the pooled effect estimates remained stable across all studies (WMD 0.01 mg/dL, 95% CI -0.50, 0.52, *N* = 15 studies, heterogeneity *P* = 0.962; Fig. 4). Subgroup analysis was performed according to regions, dose, age, baseline CRP and HbA1c, which showed no significant differences (see Additional file 1: Figure S1, Additional file 2: Figure S2; Additional file 3: Figure S3, Additional file 4: Figure S4 and Additional file 5: Figure S5). However, serum concentrations of CRP were significantly reduced in subgroup analysis stratified by diabetes duration (− 0.23 mg/L, 95% CI, − 0.41 to − 0.05) (see Additional file 6: Figure S6). Finally, there was no publication bias according to Begg’s test (*p* = 0.293) and Egger’s test (*p* = 0.332) among the seven sitagliptin studies (Fig. 5).

## Discussion

In this meta-analysis, impact of DPP-4i on CRP concentrations was evaluated among 1607 participants with T2DM. Pooled analysis from extracted studies indicated that DPP-4i significantly decreased serum CRP concentrations compared to placebo. No significant effect on CRP was observed with treatment of DPP-4i compared to that of traditional antidiabetic drugs.

This meta-analysis revealed that vildagliptin and sitagliptin had a beneficial influence on reducing serum CRP concentrations. Mechanisms of which DPP-4i reduced serum CRP potentially explained these results. Soluble DPP4 has previously been identified as cytokine related to obesity. In vitro experiments reveal that soluble DPP4 could induce inflammatory reaction by activating MAPK and NF-κB pathway via protease-activated receptor 2-dependent mechanisms [36]. Soluble DPP4 is released from cell-surface to the circulation, increasing expression and secretion of pro-inflammatory cytokines like monocyte chemoattractant protein-1 (MCP-1) and interleukin-6 (IL-6). On the other hand, gemigliptin, one of the DPP-4i, improves lipopolysaccharide (LPS)-mediated pro-inflammatory effects in vascular endothelial cells by attenuating NF-κB and JNK signaling in an Akt-AMPK-dependent pathway [37]. Besides, evidence from animal studies also supported our results, in which DPP-4i presented favorable anti-inflammatory effect against diabetes and atherosclerosis via targeting proteins participating in inflammatory pathways [38].

It has been recognized that inflammation plays a key role in endothelial dysfunction in diabetes and atherosclerosis. Fortunately, sitagliptin has been demonstrated to improve endothelial dysfunction by increasing the release of endothelial progenitor cells (EPCs) through augmentation of number of stromal cell-derived factor-1α (SDF-1α) [39]. Another study also shows that sitagliptin reduces serum CRP levels via inhibiting the activation of NF-κB in T2DM, partially improving endothelial dysfunction in patients with uncontrolled diabetes and CVD [40]. Sitagliptin also inhibits the adhesion of inhibitory-κB kinase (IKKβ) to NF-κB within a short period and downregulates TLR-4 mRNA expression, indirectly resulting in a significant 20% decrease in CRP concentrations for 2 weeks. This inhibitory effect on CRP potentially indicates an antiatherogenic effect on CVD events following DPP-4i treatment [41].

In a recent meta-analysis, our team found that DPP-4i increased serum levels of adiponectin in T2DM, an adipose-specific protein which is negatively correlated with proatherogenic LDL-C and other cardiovascular risk factors for diabetes or IR [42]. In the current subgroup analysis, DPP-4i improved CRP concentrations in European participants, but not in Asian or American subjects, reasons of which might originate from different diets or other external factors. The impact of DPP-4i on CRP had not been modulated by potential variables of regions, dose, age, baseline CRP and HbA1c, except for diabetes duration. Further researches would have to explore the exact mechanism of favorable anti-inflammatory aspects of gliptins on inhibiting the secretion of CRP. Immuno-inflammatory response correlated with hyperglycemia could promote metabolic disorder and end-stage renal disease. The effects of diabetes treatment on inflammatory reaction also suggested that patients would benefit from these drugs beyond simple control of glucose homeostasis [43].

Given that CVD is a major cause of mortality in T2DM, CRP has been proven to be a better predictor for coronary heart disease than other inflammatory markers, such as tumor necrosis factor alpha (TNF-α) and IL-6. It is crucial to explore the safety and efficacy of current glucose-lowering agents, especially, the novel class of DPP-4i. It was the first meta-analysis that assesses and demonstrates the impact of chronic treatment with DPP-4i on inflammatory markers that are known to be associated with T2DM and obesity. Patients from Europe and Asia mostly received vildagliptin and sitagliptin treatment. Adverse events and safety were evaluated and recorded in Table 3. DPP-4i were well tolerated and associated with low risks of gastrointestinal disorders and hypoglycemia (FPG < 60 mg/dL) when used alone, and all of adverse events were mild to moderate and transient. Episodes of hypoglycemia were commonly associated with precipitating factors, such as skipped meals, delay in eating and enhanced activity [23]. DPP-4i limited the breakdown of gastrointestinal hormones of glucagon-like peptide 1 (GLP-1) and glucose-dependent insulinotropic peptide (GIP), which might partially result in gastrointestinal discomfort. None of records produced severe adverse events, results of which were in line with other analysis [44, 45].

**Table 3:** Adverse events reported in the extracted studies

Study, year	Group: dose(n)	Hypoglycemia (FPG < 60 mg/dL) (n)	Gastrointestinal disorder (n)	Upper respiratory tract infection (n)	ALT > 3 times ULN (n)	Vomiting(n)	Nausea(n)	Diarrhea(n)
Derosa, 2012a [20]	sita:100 mg + met:2500 mg(91)	0	1	NS	NS	2	0	3
	met:2500 mg(87)	0	2	NS	NS	1	1	0
Asahara, 2013 [21]	sita:50 mg(24)	NS	NS	NS	NS	NS	NS	NS
	pla:50 mg(24)	NS	NS	NS	NS	NS	NS	NS
Suzuki, 2014 [22]	sita:50 mg(16)	NS	NS	NS	NS	NS	NS	NS
	lira:0.9 mg(24)	NS	NS	NS	NS	NS	NS	NS
Liu, 2013 [23]	sita:100 mg(60)	5	12	NS	1	NS	NS	NS
	piog:30 mg(60)	6	4	NS	0	NS	NS	NS
Derosa, 2010 [24]	sita:100 mg + piog:30 mg(75)	2	0	NS	NS	1	0	1
	met:850 mg + piog:30 mg(76)	0	3	NS	NS	1	2	1
Nakamura, 2014 [25]	sita:50 mg(24)	0	NS	NS	NS	NS	0	0
	vog:0.6 mg(31)	NS	NS	NS	NS	NS	1	1
Derosa, 2012b [26]	vild:100 mg + met:2500 ± 500 mg(84)	0	0	NS	NS	2	2	0
	Pla + met:2500 ± 500 mg(83)	0	2	NS	NS	0	0	3
Strozik, 2014a [27]	vild:100 mg + met:1500 mg(15)	0	3	NS	NS	NS	NS	NS
	met:1500 mg(13)	0	0	NS	NS	NS	NS	NS
Zografou, 2015 [28]	vild:50 mg + met:850 mg(32)	NS	NS	0	NS	NS	NS	NS
	met: 850 mg(32)	NS	NS	1	NS	NS	NS	NS
Derosa, 2014 [29]	vild:100 mg(86)	0	NS	NS	NS	NS	NS	NS
	glim:6 mg(70)	8	NS	NS	NS	NS	NS	NS
Kim, 2017 [30]	vild:50 mg(14)	NS	NS	NS	NS	NS	NS	NS
	piog:15 mg(11)	NS	NS	NS	NS	NS	NS	NS
Mita, 2015 [31]	alo:25 mg(172)	5	NS	NS	NS	NS	NS	NS
	con(169)	6	NS	NS	NS	NS	NS	NS
Boer, 2017 [32]	lin:5 mg(22)	NS	NS	NS	1	NS	NS	NS
	pla(22)	NS	NS	NS	0	NS	NS	NS
Yamada, 2017 [33]	sig:50 mg(55)	NS	NS	NS	NS	NS	NS	NS
	con(60)	NS	NS	NS	NS	NS	NS	NS
Koren, 2012 [34]	sita:100 mg(20)	1	NS	NS	NS	NS	NS	NS
	glib:5 mg(20)	14						
Nogueira, 2014 [35]	sita:100 mg(18)	NS	NS	NS	NS	NS	NS	NS
	insu:11 ± 6.7(17)							

*Abbreviations*: *n* Number of participants per group, *sita* Sitagliptin, *vild* Vildagliptin, *alo* Alogliptin, *metf* Metformin, *pla* Placebo, *con* Conventional treatment, *lira* Liraglutide, *piog* Pioglitazone, *vog* Voglibose, *glim* Glimepiride, *FPG* Fasting plasma glucose, *lin* Linagliptin, *glib* Glibenclamide, *cos* Chitosan oligosaccharide, *NS* Not stated

The present study is the first meta-analysis evaluating the impact of DDP-4i on serum CRP concentrations in T2DM patients. It is suggested that DPP-4i possess favorable effect against atherosclerosis and CVD events. It also provides insights into the therapeutic implications in diabetic-related atherosclerotic disease in humans for the potentially protective effects on inflammation. Secondly, results suggest that CRP might potentially serve as cardiovascular biomarker in T2DM. Thirdly, subgroup analysis has been performed to explore the influence of diabetes duration, race, dose and age.

However, this meta-analysis also has some limitations. Firstly, smaller number of patients and smaller number of trials were identified, and these studies only reported relatively short-term effect of DPP-4i on CRP levels. Secondly, only studies published in English were extracted, which inevitably resulted in potential publication bias. Finally, some heterogeneity was present in some of pooled results, although measures had been taken to overcome it by performing a sensitivity analysis.

## Conclusions

DPP4i could effectively reduce serum CRP concentrations, which might prevent exacerbation of cardiovascular events in diabetes progress. Long-term effects and cardiovascular endpoints should be studied to better guide clinicians to integrate resolutions in patients with T2DM.

## Additional files

Additional file 1: **Figure S1.** Forest plot for the impact of DDP-4i treatment versus active comparator on serum concentrations of CRP in subgroups of trials with regions of Asian and European. (TIF 522 kb)

Additional file 2: **Figure S2** Forest plot for the impact of DDP-4i treatment versus active comparator on serum concentrations of CRP in subgroups of trials with agents of low and normal dose. (TIF 545 kb)

Additional file 3: **Figure S3.** Forest plot for the impact of DDP-4i treatment versus active comparator on serum concentrations of CRP in subgroups of trials with ages of <= 60 years and > 60 years. (TIF 549 kb)

Additional file 4: **Figure S4.** Forest plot for the impact of DDP-4i treatment versus active comparator on serum concentrations of CRP in subgroups of trials with baseline CRP levels of <= 2 mg/L and > 2 mg/L. (TIF 775 kb)

Additional file 5: **Figure S5.** Forest plot for the impact of DDP-4i treatment versus active comparator on serum concentrations of CRP in subgroups of trials with HbA1c levels of <= 8.0% and > 8.0%. (TIF 773 kb)

Additional file 6: **Figure S6.** Forest plot for the impact of DDP-4i treatment versus active comparator on serum concentrations of CRP in subgroups of trials with diabetes durations of <= 12 months and > 12 months. (TIF 808 kb)

## Data Availability

All data generated or analyzed during this study are included in this published article [and its supplementary information files].

## Abbreviations

CI: Confidence intervals
CRP: C-reactive protein
CVD: Cardiovascular disease
DPP-4i: Dipeptidyl peptidase-4 inhibitors
EPCs: Endothelial progenitor cells
GIP: Glucose-dependent insulinotropic peptide
GLP1: Glucagon-like peptide 1
IKKβ: Inhibitory-κB kinase
IL-6: Interleukin-6
IR: Insulin resistance
LPS: Lipopolysaccharide
MCP-1: Monocyte chemoattractant protein-1
RCTs: Randomized controlled trials
SD: Standard deviations
SDF-1α: Stromal cell-derived factor-1α
T2DM: Type 2 diabetes mellitus
TZDs: Thiazolidinediones
WMD: Weighted mean difference
