# Supplementary figures and images for: Effect of dipeptidyl-peptidase-4 inhibitors on C-reactive protein in patients with type 2 diabetes: a systematic review and meta-analysis
Source: Lipids Health Dis. 2019 Jun 18;18:144. doi: 10.1186/s12944-019-1086-4 (PMC6580696; doi:10.1186/s12944-019-1086-4)

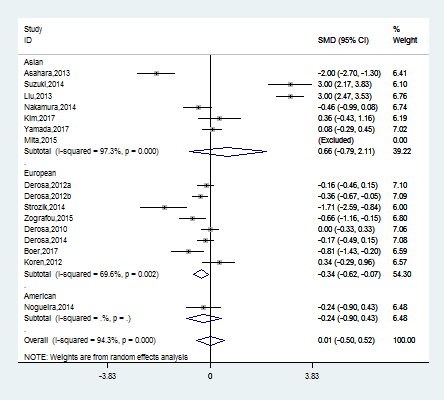

Supplement: Supplementary file 1 — Figure S1. Forest plot for the impact of DDP-4i treatment versus active comparator on serum concentrations of CRP in subgroups of trials with regions of Asian and European. (TIF 522 kb) [file 12944_2019_1086_MOESM1_ESM.tif]

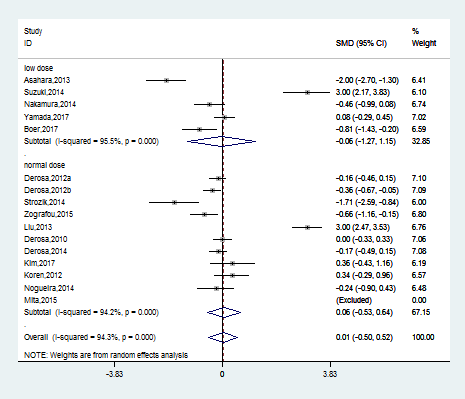

Supplement: Supplementary file 2 — Figure S2 Forest plot for the impact of DDP-4i treatment versus active comparator on serum concentrations of CRP in subgroups of trials with agents of low and normal dose. (TIF 545 kb) [file 12944_2019_1086_MOESM2_ESM.tif]

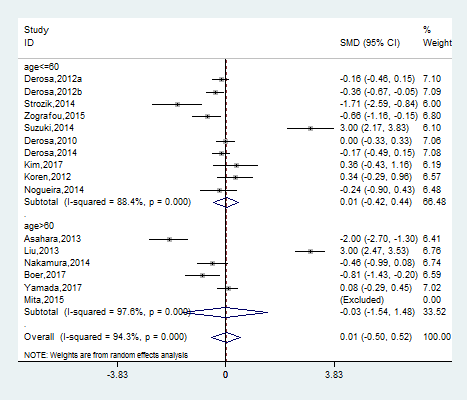

Supplement: Supplementary file 3 — Figure S3. Forest plot for the impact of DDP-4i treatment versus active comparator on serum concentrations of CRP in subgroups of trials with ages of <= 60 years and > 60 years. (TIF 549 kb) [file 12944_2019_1086_MOESM3_ESM.tif]

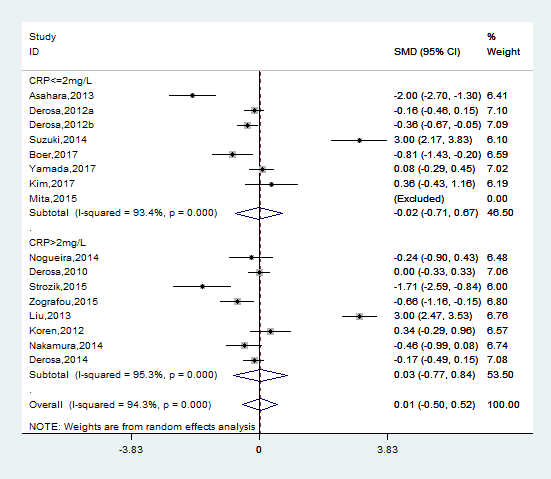

Supplement: Supplementary file 4 — Figure S4. Forest plot for the impact of DDP-4i treatment versus active comparator on serum concentrations of CRP in subgroups of trials with baseline CRP levels of <= 2 mg/L and > 2 mg/L. (TIF 775 kb) [file 12944_2019_1086_MOESM4_ESM.tif]

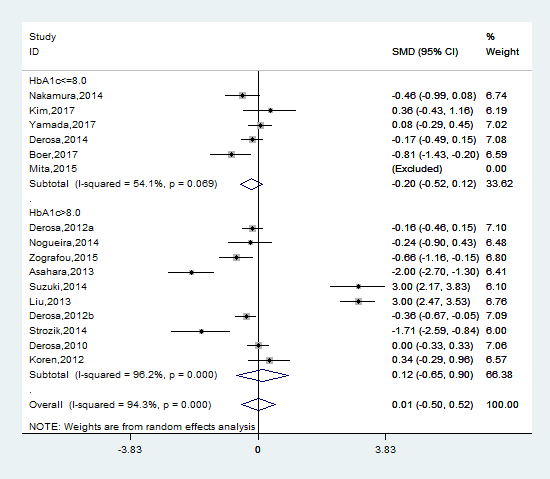

Supplement: Supplementary file 5 — Figure S5. Forest plot for the impact of DDP-4i treatment versus active comparator on serum concentrations of CRP in subgroups of trials with HbA1c levels of <= 8.0% and > 8.0%. (TIF 773 kb) [file 12944_2019_1086_MOESM5_ESM.tif]

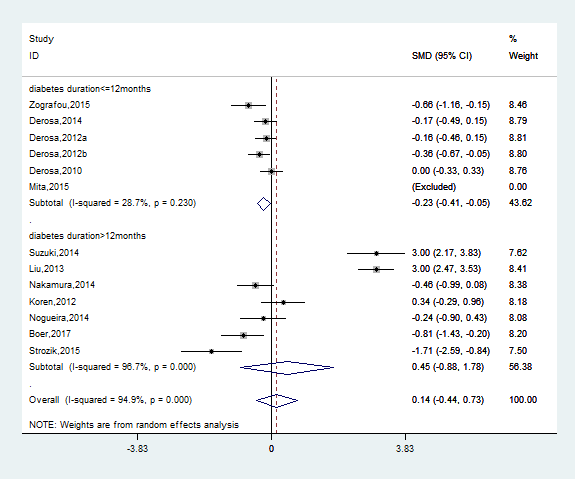

Supplement: Supplementary file 6 — Figure S6. Forest plot for the impact of DDP-4i treatment versus active comparator on serum concentrations of CRP in subgroups of trials with diabetes durations of <= 12 months and > 12 months. (TIF 808 kb) [file 12944_2019_1086_MOESM6_ESM.tif]
